# Comparison of Different Ranking Methods in Protein-Ligand Binding Site Prediction

**DOI:** 10.3390/ijms13078752

**Published:** 2012-07-16

**Authors:** Jun Gao, Qi Liu, Hong Kang, Zhiwei Cao, Ruixin Zhu

**Affiliations:** 1 College of Life Science and Biotechnology, Tongji University, Shanghai 200092, China; E-Mails: jungao@shmtu.edu.cn (J.G.); qiliu@tongji.edu.cn (Q.L.); kangh67@hotmail.com (H.K.); 2 College of Information Engineering, Shanghai Maritime University, Shanghai 201306, China; 3 Institute for Advanced Study of Translational Medicine, Tongji University, Shanghai 200092, China; 4 School of Pharmacy, Liaoning University of Traditional Chinese Medicine, Dalian 116600, China

**Keywords:** ranking aggregation, protein-ligand binding site, prediction

## Abstract

In recent years, although many ligand-binding site prediction methods have been developed, there has still been a great demand to improve the prediction accuracy and compare different prediction algorithms to evaluate their performances. In this work, in order to improve the performance of the protein-ligand binding site prediction method presented in our former study, a comparison of different binding site ranking lists was studied. Four kinds of properties, *i.e.*, pocket size, distance from the protein centroid, sequence conservation and the number of hydrophobic residues, have been chosen as the corresponding ranking criterion respectively. Our studies show that the sequence conservation information helps to rank the real pockets with the most successful accuracy compared to others. At the same time, the pocket size and the distance of binding site from the protein centroid are also found to be helpful. In addition, a multi-view ranking aggregation method, which combines the information among those four properties, was further applied in our study. The results show that a better performance can be achieved by the aggregation of the complementary properties in the prediction of ligand-binding sites.

## 1. Introduction

In most cellular processes, proteins interact with many other molecules to perform their biological functions. The successful identification of ligand-binding sites on protein surfaces is generally the starting point for the annotation of protein function and drug discovery. In addition, as a result of various structural genomic projects performed, structural information of proteins with little or no functional annotations is increasing exponentially. However, in most cases, protein-ligand complex structures are not easily experimentally accessible, which leads to the demand of *in silico* methods to serve as an alternative [1,2]. Fortunately, it has been proven that the prediction of binding sites using computational methods is efficient and powerful compared to *in vivo* approaches, and several computational methods have been presented in this area [3,4]. However, research in this area is clearly in an infant stage and there still remain many issues to be solved and improved.

To predict the potential binding site, several computational methods have been developed. Briefly, these algorithms can be divided into three categories, *i.e.*, (1) purely geometry-based methods, which follow the assumption that the protein-ligand binding sites are generally located at crevices on the protein surface or cavities in the protein. Methods falling in this category include POCKET [5], LIGSITE [6], PASS [7], SURFNET [8], and PocketPicker [9] *etc*.; (2) energetic-based methods, which coat the protein surface with a layer of probes to calculate van der Waals interaction energies between the protein and probes. As an example, Q-SiteFinder [10] is a classical tool falling in this category; (3) knowledge based methods, which includs various statistical methods [11], machine learning methods [12] and similarity comparison methods. Besides, a part of them predict protein-ligand binding sites by searching for clusters or patterns of the conserved residues [13,14].

Generally speaking, a computational method for binding site prediction has to consider several challenging issues: (1) Identification of candidate protein-ligand binding sites [5–17], which relate to delimit cavities or pockets at the protein surface that are likely to bind molecules; (2) ranking binding sites according to their likeliness to accept a molecule, since there are often several presumed binding sites that can be predicted on a protein surface, and it is necessary to derive an approach to characterize and rank them to select the more relevant ones [18]; (3) induced fit, which may enhance the fidelity of molecular recognition in the presence of competition and noise via conformational proofreading mechanism [19]. In this study, we focus primarily on the ranking of binding sites. It is said that the largest pocket tends to frequently correspond to the observed ligand-binding site [20]. Based on this assumption, most prediction methods rank the candidate sites according to the pocket size. Nevertheless, different studies have also tried to solve this ranking problem from other perspectives [16,21,22].

Our former work for binding site prediction is based on the integration of sequence conservation information with geometry-based cleft identification. In this study, in order to improve the performance of our work and investigate the contribution of different ranking methods in the prediction of protein-ligand binding sites, five ranking methods (pocket size, distance from the protein centroid, sequence conservation, number of hydrophobic residues, multi-view method) involving four properties have been tested. The results show that (1) if only one property is considered, the use of sequence conservation information helps ranking the pockets best; and (2) the innovative multi-view method, which integrates complementary properties such as pocket size and distance from the protein centroid, can achieve a better performance than if only one individual property is considered.

## 2. Results and Discussion

### 2.1. Individual Property Comparison

For the bound and unbound/bound test sets, 17 pockets were predicted for each protein on average with our geometry-based site finding method. The TOP1 and TOP3 accuracy differs for different ranking methods. The accuracy of the TOP 1 and TOP 3 in different individual property prediction ranking lists is listed in Table 1. A geometry-based method, SURFNET [8], with its own ranking algorithm is also tested for comparison. It is shown that ranking that presumes binding sites according to conservation score achieves the best performance with a 59% success rate in the top 1 prediction, which means that almost 124 of the 210 proteins in the bound test set are correctly predicted. Ranking with the criterion of “volume and distance from the protein centroid” (shown in the “Distance” column) also performs with better results, which may indicate that the size and the depth of the binding site could be helpful in ligand binding site prediction. However, we found that ranking according to the hydrophobic attribute does not deliver the expected results. We explain this by the fact that the description of hydrophobic properties in our study may be too simple.

### 2.2. Ranking Aggregation from a Multi-View Perspective

**Figure 1:**
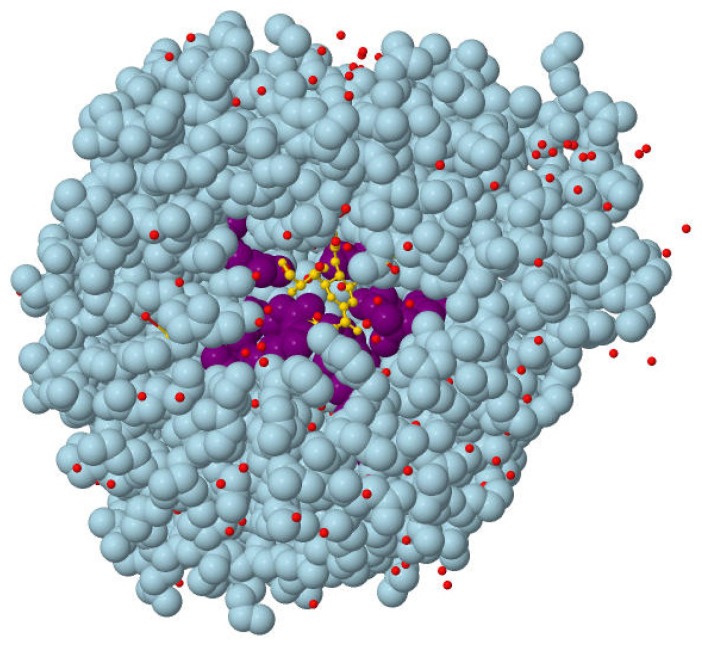
The surface position of Pocket 9 in protein structure. PDB ID: 2SIM. (Red points: water molecule; Light blue: the whole protein; Golden: molecular ligand; Purple: predicted binding site constituted by amino acids).

In some cases the conservation profiles of proteins are not easily accessible, which may make it impossible to rank presumed binding sites by conservation score. In addition, there is an urgent need for developing an efficient approach to fully integrate various complementary ranking lists from a comprehensive multi-view perspective. Thus in our study, an innovative ranking aggregation method is further applied to address these problems. We integrate the ranking lists of different properties like the combination of “binding site size” and “the distance from the protein centroid”. The corresponding results are listed in Table 2. It is shown that after the ranking aggregation, most of the success rates are improved remarkably and some of them are comparable to the conservation ones. These results indicate that the combination of different individual complimentary properties will generally improve the prediction success rate. In addition, “Volume plus Distance” is found to be an alternative to “Conservation” when proteins with no conservation profiles are predicted. An example (PDB: 2SIM [23]) for such a kind of ranking aggregation is presented in Table 3. It can be seen that the ordering of the correctly predicted binding sites (*Pocket 9) is promoted after ranking aggregation, which leads to the improvement of the TOP 1 success rate. In Figure 1, the surface position of Pocket 9 is visualized with Jmol [24]. However, it is worth noting that when two or more properties that are not complementary are used, such as the information of volume and conservation, the final success rate probably does not show any improvement.

In summary, our study has not only validated the significance of sequence conservation in ligand binding site prediction, but also indicated the usefulness of the size and depth of the binding site in the ranking of binding sites. Furthermore, rather than only considering one property, an innovative multi-view ranking method was applied, which could achieve a much better performance for binding site prediction.

## 3. Methods

**Figure 2:**
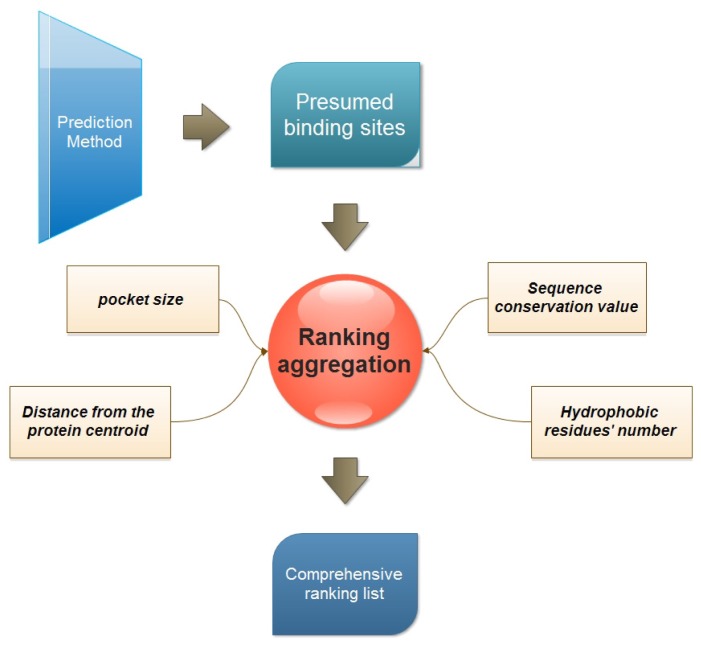
The concept of multi-view ranking aggregation.

Our study relies on a new protein-ligand binding site prediction method introduced in our previous work. It is based on the integration of geometry and sequence conservation information [4]. An overview of the ranking study is presented in Figure 2.

### 3.1. Four Properties Used for Ranking

The four properties for the ranking of binding sites are calculated as follows:

1. Pocket size. This is one of the most popular ranking properties. In this study, the volume of every presumed binding site is calculated with the Qhull program [25].
2. Distance of binding site from the protein centroid. This property is considered to reflect the depth of a presumed binding site. And the distance is defined as the Euclidian distance between the protein centroid and the geometric center of the presumed binding site.
  (1) $$d=\sqrt{{\left(x_{b}-x_{p}\right)}^{2}+{\left(y_{b}-y_{p}\right)}^{2}+{\left(z_{b}-z_{p}\right)}^{2}}$$
  where (*x_b_*, *y_b_*, *z_b_*) is the coordination of the predicted binding site center, and (*x_p_*, *y_p_*, *z_p_*) is the center of the protein.
3. Sequence conservation value. The sequence conservation information is achieved by the ConSurf-DB [26], which provides the pre-calculated evolutionary conservation profiles for proteins with known structures in the PDB. In ConSurf-DB, every residue in every corresponding protein is evaluated with a normalized conservation score so that its average over all residues is zero and the standard deviation is one. Low (negative) scores indicate the conserved positions while the high scores indicate the variable ones. In our study, the candidate binding sites are ranked according to the conservation score of all residues in the same binding site.
4. The number of hydrophobic residues. Due to the importance of hydrophobicity in protein-ligand binding sites [27,28], the number of hydrophobic residues in each presumed binding site is also calculated. The hydrophobic residues include ALA, VAL, LEU, ILE, PRO, PHE, TRP and MET. The following equation is used to calculate hydrophobic residues:
  (2) $$N_{H}=\sum n_{i},\;i\in \{ALA,VAL,LEU,ILE,PRO,PHE,TRP,MET\}$$

### 3.2. Multi-View Ranking Aggregation

The complementary properties listed above might be helpful in ranking presumed binding sites. Such an innovative ranking aggregation method was also applied in our previous study [29]. It is based on the equalitarian philosophical paradigm to seek a consensus list among individual ranking lists. Before defining the two distance measures, some necessary notations should be introduced. Let *M_i_*(1), ···, *M_i_*(*k*) be the scores associated with the ordered list *L_i_*, where *M_i_*(1) is the best score, *M_i_*(2) is the second best one, and so on. Let *r_Li_*(*A*) be the rank of A in the list *L_i_* if A is within the top *k*, and otherwise equal to *k* + 1. The distance between two ranking lists can be defined as:

(3) $$d(L_{i},L_{j})=\sum_{t\in L_{i}\cup L_{j}}|r^{L_{i}}(t)-r^{L_{j}}(t)|$$

which is also named the Spearman’s footrule distance [30]. *r^L_j_^* *(t)* in equation (3) indicates the position of element *t* in the ordered list *j*.

In order to discover a comprehensive ranking list that would also be as close as possible to all the given ranking lists, an optimization function is defined:

(4) $$\delta^{*}=\text{arg }(\text{min}\{\Phi (\delta )\})$$

(5) $$\Phi (\delta )=\sum_{i=1}^{m}W_{i}d(\delta ,L_{i})$$

where *W_i_* is the importance weight of ranking list *L_i_*. It is set to one in our study as we treat the four properties equally. Parameter *d*, which is calculated according to Spearman distance, represents the distance between the “comprehensive list” δ^*^ and *L_i_*. The goal of the ranking aggregation is to find δ^*^ which minimizes the total distance between the “comprehensive list” and every ranking list. To accomplish this goal, the Cross-Entropy method (CE) [31] is used here, which is a general Monte Carlo approach for multi-extremum optimization. The CE algorithm requires users to set a number of parameters. It is recommended that the number of samples *N* for each stage is set to at least 10 *k*^2^, and the rarity parameter *ρ* in the sampling stage of CE [31] used to update the cell probabilities is set to 0.01 when *N* is relatively large, and 0.1 when *N* is small (less than 100). All data are aggregated under *R* statistical environment with the *RankAggreg* package.

### 3.3. Test Dataset and Evaluation of the Pocket Prediction

In this study, two datasets, *i.e.*, the 210 bound structures and 48 unbound/bound structures, which are used to evaluate the LIGSITE^csc^ [16] algorithm are also used as a kind of unbound/bound and bound test set. To assess the quality of binding-site predictions, a standard evaluation method presented previously [4,6,9,16] is applied, which defines a prediction to be a met, if the geometric center of the presumed pocket lies within 4 Å to any atom of the ligand. Predictions that do not meet this criterion are excluded in the calculation of prediction success rates.

We also used another evaluation measurement, *i.e.*, the Matthews Correlation Coefficient [32] (MCC) as a comparison. For each protein, residue predictions were classified as true positives (TP: correctly predicted binding site residues), true negatives (TN: correctly predicted nonbinding site residues), false negatives (FN: incorrectly predicted as nonbinding site residues), false positives (FP: incorrectly predicted as binding site residues). The MCC was computed using Equation 6:

(6) $$MCC=\frac{TP\times TN-FP\times FN}{\sqrt{\left(TP+FP)\times (TP+FN)\times (TN+FP)\times (TN+FN\right)}}$$

For the bound and unbound/bound test sets, the MCC score for each protein can be calculated with a certain prediction method. In our implementation, different score can be calculated for different ranking methods. To determine the significant differences between different ranking methods as well as their combinations, the one-sided Wilcoxon signed ranked sum test is used based on MCC scores for each protein. The statistical evaluation is performed using *R* (version 2.15.0).

For the 210 bound structures, the evaluation is very straightforward and we will follow the above described routing procedure. For the unbound/bound dataset, the Biojava development package [33] is first used for the alignment of all the structures, and the ligands in the bound structures are mirrored to the corresponding unbound structures. Finally the predictions are performed for the unbound structures and then they are checked against the bound structures.

## 4. Conclusions

The prediction of protein-ligand binding sites has great significance for protein function annotation and computer-aided drug design. Besides the binding site identification, the binding sites’ ranking according to their likeliness to accept a molecule is also an important and challenging issue. In order to improve the findings of our previous work, this paper represents an initial effort to study the contribution of different ranking methods to protein-ligand binding site prediction. Five ranking methods (pocket size, distance from the protein centroid, sequence conservation, number of hydrophobic residues, multi-view ranking aggregation) have been tested in our study. The results show that when only one property is considered, the use of sequence conservation information helps ranking the pockets best. In addition, pocket size and depth can also serve as important attributes. Moreover, it is also proven that ranking aggregation which involves complementary properties can obtain a better performance than that of individual properties. This finding not only supports the findings of our previous work, but also provides useful suggestions for other related binding site identification studies.

## Figures and Tables

**Table 1:** Prediction success rate presented by different ranking methods.

	Bound			Unbound/bound		
		
Methods	TOP1	MCC for TOP1	TOP3	TOP1	MCC for TOP1	TOP3
Conservation score	59%	0.53	73%	57	0.53	72
Distance	48%	0.53	66%	56	0.53	70
Volume	47%	0.50	69%	44	0.53	59
Hydrophobic	39%	0.51	62%	30	0.51	48
SURFNET (Control)	42%	~	57%	~	~	~

**Table 2:** Prediction success rate of ranking aggregation.

	Bound			Unbound/bound		
		
Methods	TOP1	MCC * for TOP1	TOP3	TOP1	MCC for TOP1	TOP3
CON + DIS	57%	0.52	74%	61	0.53	74
VOL + DIS	52%	0.51	73%	54	0.53	74
CON + VOL	52%	0.52	72%	48	0.54	65
VOL + HYDRO	46%	0.50	67%	39	0.53	61
DIS + HYDRO	47%	0.51	68%	44	0.49	63
CON + HYDRO	53%	0.51	70%	39	0.53	61
DIS + CON + HYDRO	53%	0.50	72%	48	0.51	67
VOL + CON + HYDRO	51%	0.52	71%	41	0.55	63
VOL + DIS + HYDRO	50%	0.52	71%	46	0.50	67
VOL + DIS + CON	54%	0.51	73%	52	0.53	74
VOL + DIS + CON + HYDRO	53%	0.52	72%	48	0.53	67

* The one-sided Wilcoxon signed ranked sum test is used based on the Matthews Correlation Coefficient (MCC) scores for each protein. The *p* values for the comparison of different methods are listed in the Supporting Information (Table S1 for bound test set, S2 for unbound/bound test set).

**Table 3:** Part of results obtained for different ranking methods, which include volume (VOL), distance of presumed binding sites from the protein centroid (DIS), rank aggregation (REG) for VOL and DIS, and conservation score (CONS).

Rank	VOL	DIS	REG	CONS
1	Pocket 0	Pocket 12	*Pocket 9	*Pocket 9
2	*Pocket 9	*Pocket 9	Pocket 0	Pocket 5
3	Pocket 5	Pocket 0	Pocket 10	Pocket 0
4	Pocket 10	Pocket 7	Pocket 12	Pocket 2

* Pocket 9 corresponds to the observed binding site.
